# High revision rate of metal-backed glenoid component and impact on the overall revision rate of stemless total shoulder arthroplasty: a cohort study from the Danish Shoulder Arthroplasty Registry

**DOI:** 10.2340/17453674.2024.41014

**Published:** 2024-07-17

**Authors:** Marc R K NYRING, Bo S OLSEN, Steen L JENSEN, Jeppe V RASMUSSEN

**Affiliations:** 1 Department of Orthopedic Surgery, Herlev and Gentofte Hospital, Hellerup; 2 Department of Orthopedic Surgery, Aalborg University Hospital, Aalborg, Denmark

## Abstract

**Background and purpose:**

There is controversy regarding the results of stemmed and stemless total shoulder arthroplasty (TSA) used for osteoarthritis. Therefore, we aimed to compare revision rates of stemmed and stemless TSA and to examine the impact of metal-backed glenoid components.

**Methods:**

We included all patients reported to the Danish Shoulder Arthroplasty Register from January 1, 2012 to December 31, 2022 with an anatomical TSA used for osteoarthritis. Primary outcome was revision (removal or exchange of components) for any reason.

**Results:**

3,338 arthroplasties were included. The hazard ratio for revision of stemless TSA adjusted for age and sex was 1.83 (95% confidence interval [CI] 1.21–2.78) with stemmed TSA as reference. When excluding all arthroplasties with a metal-backed glenoid component, the adjusted hazard ratio for revision of stemless TSA was 1.37 (CI 0.85–2.20). For the Eclipse stemless TSA system, the adjusted hazard ratio for revision of a metal-backed glenoid component was 8.75 (CI 2.40–31.9) with stemless Eclipse with an all-polyethylene glenoid component as reference.

**Conclusion:**

We showed that the risk of revision of stemless TSAs was increased and that it was related to their combination with metal-backed glenoid components.

In patients with end-stage osteoarthritis and an intact rotator cuff, the stemmed total shoulder arthroplasty (TSA) has been the treatment of choice for many decades [1] with good postoperative results [2-5], but in recent years, the use of the stemless TSA has increased [6]. The stemless TSA design leads to several potential advantages, such as shorter surgical time [7,8] and reduced blood loss [7,9]. Another advantage of the stemless TSA is that the inclination angle is not predetermined, giving the surgeon the possibility of more precise and individual cutting of the humeral head [10]. Finally, due to the canal-preserving design, a revision procedure is theoretically more feasible [10].

Several studies have documented equally good results comparing stemmed and stemless TSA [6,11-14]. However, in the most recent annual report from the Danish Shoulder Arthroplasty Register (DSR) [15], a comparison of stemmed and stemless TSA indicated a significantly higher revision rate for stemless TSA. This result from the annual DSR report conflicts with the previous publications. Additionally, it appeared in the annual report from the DSR that stemless TSA had more often been used in combination with metal-backed glenoid components than stemmed TSA. Therefore, we found it of relevance to conduct a more detailed comparison of stemmed and stemless TSA.

The primary aim was to compare the revision rates of stemmed and stemless TSA for glenohumeral osteoarthritis. The secondary aim was to compare the revision rates of stemless and stemmed TSA in combination with a metal-backed or an all-polyethylene glenoid component.

## Methods

### Study design

The study is an observational registry cohort study based on prospectively collected data from the DSR. The guidelines for reporting observational studies (e.g., the STROBE statement) were followed.

### Data source

In 2004, the DSR was established as a government-financed registry. In both public and private hospitals, it is mandatory for the surgeon to report both primary shoulder arthroplasties and revision procedures at the time of surgery [5]. The surgeon reports patient-specific data such as sex, date of birth, diagnosis, and previous surgeries and surgical data such as surgery time, brand, implant characteristics, and approach. In case of revision, the operating surgeon reports the type of revision and the reason for it. In the DSR, revision is defined as removal, exchange, or addition of any component [16]. Reoperations without removal of components are not registered in the DSR. The completeness of data collection has been above 92% in all years during the study period [15] with data from the Danish National Patient Registry as the reference [5].

### Population

Until 2012, only a few stemless TSA had been reported to the DSR. Therefore, we included all patients reported to the DSR from January 1, 2012 to December 31, 2022 with an anatomical TSA used for glenohumeral osteoarthritis. Patients with hemiarthroplasties, reverse shoulder arthroplasties, mini stems, and patients with primary diagnoses other than osteoarthritis were excluded. Mini-stem arthroplasties are characterized by a small stem that is inserted into the bone canal, while the stemless arthroplasty relies on metaphyseal fixation alone. We included all bilateral cases, as previous studies have shown that the presence of bilaterality does not affect the result significantly in arthroplasty registry studies [17,18].

### Outcome

All revision arthroplasties of either or both of the components were identified and linked to the primary arthroplasty using the unique Danish central personal registration number [6]. The individual patient time at risk of revision was defined as either the time from the primary procedure to the revision procedure, to death, or to the end of the inclusion period (December 31, 2022). Reoperations without removal of components are not registered in the DSR and therefore not accounted for in this study.

### Statistics

Demographic data was reported. Group differences in surgery time and time to revision were analysed using the independent samples t-test. The Kaplan–Meier method is the recommended survival function when accurate revision times are known [19]. Therefore, we used this method to calculate and illustrate the unadjusted cumulative revision rates including 95% confidence intervals (CI). In arthroplasty registry studies, death is a competing risk when calculating risk of revision. Even so, the Cox regression model is the recommended model of choice [20,21]. Therefore, we used a multivariable Cox regression model, including age and sex, to calculate the hazard ratios with 95% CI. We considered patients aged 75 or older as elderly [22]. Therefore, the patients were grouped as either <75 or ≥75 in the analyses. Patients who died during the study period were censored on the day of death; until this date, they contributed with their time at risk. Due to the assumption of proportional hazards, we compared only revision rates between the groups within the time period where this assumption was fulfilled [23]. This was secured by checking that the cumulative revision curves did not cross each other. All reported P values are 2-tailed, and the level of statistical significance was defined as P < 0.05. SPSS (IBM Corp, Armonk, NY, USA) was used for the statistical analyses and R version 4.1.0 (R Foundation for Statistical Computing, Vienna, Austria) was used for the graphical illustrations.

### Ethics, funding, data sharing, and disclosures

The Capital Region of Denmark approved the handling of data for the study, with the study number: P-2023-274. Informed consent was not required. No funding was received for the study. Data may be made available to other researchers upon request to the Danish Shoulder Arthroplasty registry given that approval from the registry can be provided. The authors have no disclosures, and complete disclosure of interest forms according to ICMJE are available on the article page, doi: 10.2340/17453674.2024.41014

## Results

**Figure 1:**
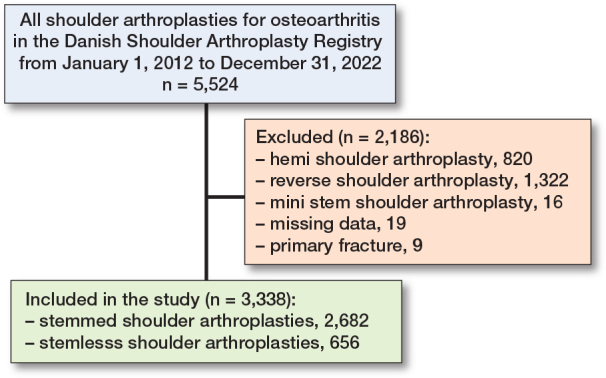
Flowchart of excluded patients.

From January 1, 2012 to December 31, 2022, 2,960 patients with 3,338 primary anatomical TSA used for glenohumeral osteoarthritis were reported to the DSR (Figure 1). 269 patients were censored because of death.

**Figure 2:**
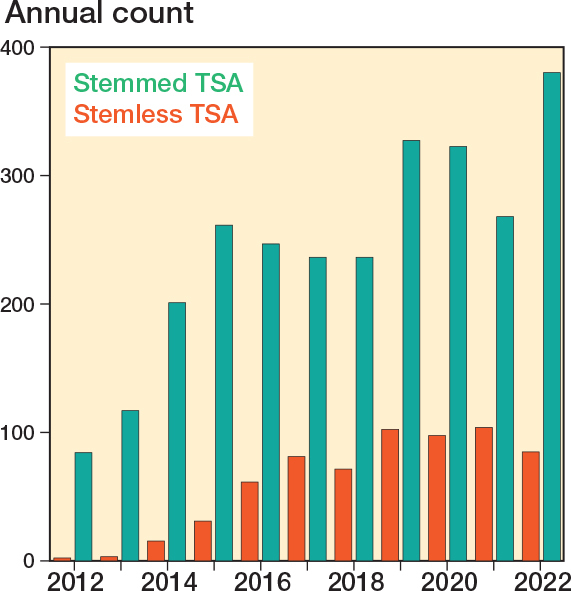
Anatomical TSAs per year in Denmark.

During the study period the use of anatomical TSA has increased to a level around 420 cases per year, with the stemless TSA accounting for approximately one-fourth of the cases in recent years (Figure 2). 2,682 stemmed TSA and 656 stemless TSA were included (Table 1). The mean surgery time was 93 minutes for stemmed TSA and 89 minutes for stemless TSA, resulting in a mean difference of 5 minutes (CI 2.5–7.2).

**Table 1:** Demographics. Values are count (%) unless otherwise specified

Variable	Stemmed (n = 2,682)	Stemless (n = 656)
Age, mean (SD)	69 (9)	66 (10)
median (IQR)	70 (64–75)	68 (61–74)
Age group		
<75	1,954 (73)	511 (78)
≥75	728 (27)	145 (22)
Female sex	1,707 (64)	363 (55)
Procedure side right	1,359 (51)	334 (51)
Deaths during the study period	234 (8.8)	41 (6.3)

32 stemless TSA (4.9%) and 77 stemmed TSA (2.9%) were revised during the study period. The adjusted hazard ratio for revision of stemless TSA was higher compared with the stemmed TSA (1.83, CI 1.21–2.78). The mean time to revision was 711 days for the stemmed TSA and 612 days for the stemless TSA, resulting in a mean difference of 99 days (CI –175 to 374).

An overview of implants, including the proportion of revisions, is presented in Table 2. The Eclipse stemless TSA system (Arthrex, Naples, FL, USA) with a metal backed glenoid component had 10 revisions in 37 cases compared with 3 in 128 cases with an all-polyethylene component. When excluding all arthroplasties with a metal-backed glenoid component (60 cases) the hazard ratio for revision of stemless TSA was reduced to 1.37 (CI 0.85–2.20) with the stemmed TSA as reference.

**Table 2:** Shoulder prosthesis models with total number and revisions

Implant	n	Revisions (%)
Stemmed		
Comprehensive Standard	641	22 (3.4)
Global Advantage	1,278	24 (1.9)
Global Unite	309	11 (3.6)
Global FX	218	9 (4.1)
Anatomical Shoulder	78	2 (2.6)
Bigliani Flatow		
All polyethylene	61	5 (8.2)
Metal backed	6	1 (17)
Lima SMR		
All polyethylene	71	1 (1.4)
Metal backed	15	2 (13)
Other		
All polyethylene	5	0 (0.0)
Metal backed	1	0 (0.0)
Stemless		
Comprehensive Nano	466	19 (4.1)
Global Icon	12	0 (0.0)
Tornier Simpliciti	11	0 (0.0)
Arthrex Eclipse		
All polyethylene	128	3 (2.3)
Metal backed	37	10 (27)
Other		
All polyethylene	0	0 (0.0)
Metal backed	1	0 (0.0)

**Figure 3:**
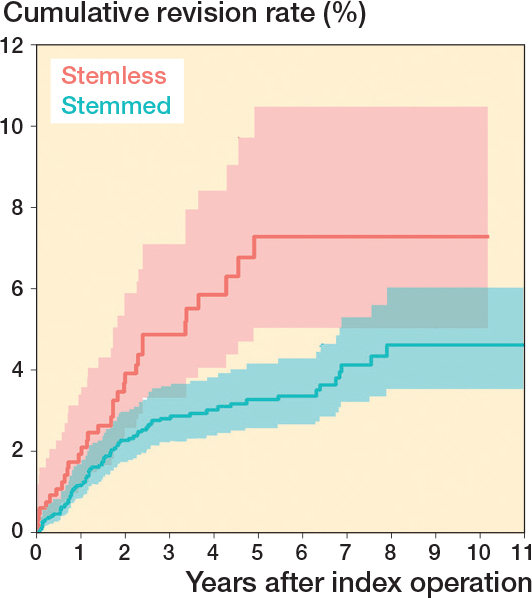
Cumulative revision rates with CIs of stemmed and stemless TSA.

**Figure 4:**
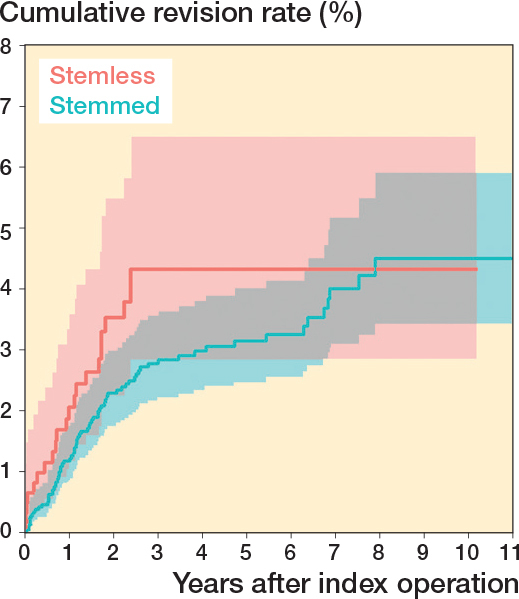
Cumulative revision rates with CIs of stemmed and stemless TSA after exclusion of arthroplasties with a metal-backed glenoid component.

The overall 5-year cumulative revision rate was 3.3% (CI 2.5–4.1) for stemmed TSA and 7.3% (CI 4.6–10.0) for stemless TSA (Figure 3). When excluding the TSA with a metal-backed glenoid component, the 5-year cumulative revision rate for the stemmed TSA was 3.1% (CI 2.3–3.9) and for the stemless TSA 4.3% (CI 2.5–6.1) (Figure 4). For both stemless and stemmed TSA, the most frequent reason for revision was rotator cuff tear (Table 3). Implant failure was reported as the reason for revision in 5 of 10 cases for the Eclipse stemless TSA with a metal-backed glenoid component; the other reasons for revision were displacement (2 cases), instability (1 case), infection (1 case), and rotator cuff tear (1 case).

**Table 3:** Reasons for revision of stemless and stemmed TSA. Values are count (%)

Reason for revision	Stemless (n = 32)	Stemmed (n = 77)
Rotator cuff tear	8 (25)	27 (35)
Aseptic loosening	2 (6.3)	15 (20)
Infection	4 (13)	10 (13)
Instability	4 (13)	4 (5.2)
Implant failure	6 (19)	4 (5.2)
Displacement	3 (9.4)	5 (6.5)
Glenoid erosion	0 (0)	2 (2.6)
Fracture	2 (6.3)	1 (1.3)
Malplacement	2 (6.3)	5 (6.5)
Other	1 (3.1)	3 (3.9)
Unknown	0 (0)	1 (1.3)

**Figure 5:**
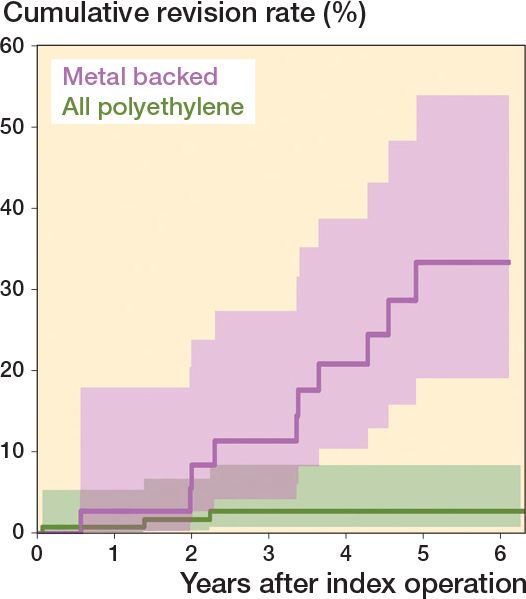
Cumulative revision rates with CIs of the Eclipse stemless TSA with a metal-backed or an all-polyethylene glenoid component.

The cumulative revision rates of the Eclipse stemless TSA were higher with a metal-backed glenoid component than with an all-polyethylene glenoid component (Figure 5) with adjusted hazard ratio for revision of 8.75 (CI 2.40–31.9).

## Discussion

We aimed to compare revision rates of stemmed and stemless TSA and to examine the impact of metal-backed glenoid components. We showed an increased risk of revision of the stemless TSA and the 5-year revision rate of stemmed and stemless TSA was 3.3% and 7.3% respectively. We showed an increased risk of revision for a specific arthroplasty brand, which previously [24] has been argued to be safe. Metal-backed glenoid components had a high revision proportion. When they were excluded from our analysis, the hazard ratio decreased from 1.83 to 1.37 and was no longer statistically significant. The Eclipse stemless TSA in combination with a metal-backed glenoid component had a 9 times higher risk of revision compared with the Eclipse stemless TSA in combination with an all-polyethylene glenoid component.

The stemless TSA might have an increased risk of revision due to loosening of the humeral implant [25], which is fixated entirely in the metaphyseal part of the humeral bone. However, so far, all previous publications have reported similar revision rates of stemmed and stemless TSA [6,11-14]. Our results contrasted with the previous findings.

Based on the proportion of revisions for each arthroplasty brand, it was obvious that the Eclipse stemless TSA in combination with a metal-backed glenoid component could influence the overall revision rate of stemless TSA. This in accordance with a study by Noschajew et al. [26], who reported 17 revisions in 25 Eclipse stemless TSA with a metal-backed glenoid component after a mean follow-up of 6 years. In addition, Magosch et al. [27] reported 6 revisions in 48 Eclipse stemless TSA with a metal-backed glenoid component, but after only a minimum 2 years’ follow-up.

Due to the low number of arthroplasties and revisions, it was not possible to draw any conclusions on the use of stemmed TSA with a metal-backed glenoid component. To our knowledge, no studies have compared the metal-backed and all-polyethylene glenoid component in combination with the Eclipse stemless TSA.

Previous randomized trials comparing the Eclipse stemless TSA system with a stemmed TSA demonstrated equally good patient-reported outcomes and similar revision rates [24,28]. In the study by Romeo et al. [28], all patients had an all-polyethylene glenoid component, which could be one reason for the low revision rate of both TSA systems. In the study by Uschok et al. [24], approximately half of the patients in both the stemless TSA group and the stemmed TSA group had a metal-backed glenoid component. They reported an overall revision rate of 13.8%, but the type of glenoid component in the revised cases was not indicated.

Previous registry-based studies have also identified metal-backed glenoid components as a risk factor for revision compared with all-polyethylene glenoid components. A study from the Australian Orthopaedic Association National Joint Replacement Registry [29] reports a hazard ratio of 4.7 for revision of TSA with metal-backed glenoid components with TSA with an all-polyethylene glenoid component as reference. This is supported by a study based on the New Zealand Joint Registry [30]. They report a 5 times higher revision rate of the TSA with metal-backed glenoid components compared with TSA with all-polyethylene components.

The reason for the high revision rate of metal-backed glenoid components is unknown. In our study, the most frequent reason for revision of a metal-backed glenoid component was implant failure. Noschajew et al. [26] reported that polyethylene wear was the most frequent reason for revision. Theoretically, the reasons for failure of the metal-backed component could be due to instability/dislocation in the transition between the metal and polyethylene part as reported by Magosch et al. [27]. It might also be related to loosening of the uncemented screw fixation.

The findings of our study support the hypothesis that metal-backed glenoid components are a risk factor for revision.

### Limitations

We were not able to adjust surgeon- and patient-related factors and especially comorbidities that may increase the risk of revision due to infection, periprosthetic fracture, polyethylene wear, or loosening [31,32]. Also, reasons for revision have not been validated.

There may be bias by indication, as there might be different reasons for choosing a stemmed or a stemless TSA. The stemless TSA is more often preferred in young patients. The reason for this is speculative but one explanation could be that the bone quality in young patients is more suitable for a stemless arthroplasty with uncemented metaphysical fixation. It might also be related to the assumption that the stemless TSA is easier to revise and therefore suitable for patients with long life expectancy. We have minimized the risk of bias by including age in the Cox regression model but differences in age are important to keep in mind when Kaplan–Meier curves are compared. There might also be different reasons for choosing an all-polyethylene or a metal-backed glenoid component. The DSR does not encompass information on glenoid morphology (e.g., Walch classification). However, it is possible that the metal-backed glenoid component with uncemented screw fixation might have been used more frequently in patients with severe bone loss. Additionally, the Eclipse stemless TSA in combination with a metal-backed glenoid component was based on few arthroplasties and few revisions, and the estimates must therefore be interpreted with caution.

In the survival analysis of registry data, death is a competing risk implying a possible overestimation of the risk of revision and more uncertain estimates the longer the follow-up time [23]. However, it has been argued that this overestimation is not relevant in arthroplasty registry studies [33] and especially not from a clinical point of view [21,23]. Therefore, the Kaplan–Meier method and the Cox regression model have been recommended in orthopedic arthroplasty registry studies [20,21]. The Kaplan–Meier method and the Cox regression model are time-dependent analyses that include the patients’ time at risk in the analyses. Therefore, these estimates are not expected to be biased by differences in follow-up time [19]. It is, however, important to keep in mind that the short follow-up time especially of the stemless TSA leads to more uncertain mid- and long-term estimates.

### Strengths

The registry-based design also entails important strengths. The large population facilitates analyzes and sub-analyzes on different combinations of humeral and glenoid components. Additionally, the dataset is based on the entire population of Denmark, including both low- and high-volume centers, making the results generalizable with high external validity.

### Conclusions

We showed that the risk of revision for stemless arthroplasties was increased and that it may be related to their combination with metal-backed glenoid components.

## Notes

MN: study design, data analysis and draft of the manuscript. BO, SJ and JR: study design and writing of the manuscript.

Handling co-editors: Li Felländer-Tsai and Philippe Wagner

*Acta* thanks Hanna Cecilia Björnsson Hallgren and Björn Salomonsson for help with peer review of this manuscript.
